# Evaluation of the Use of Phosphatidic Acid in the Diet on Growth Performance and Breast Meat Yield in Broilers

**DOI:** 10.3390/ani8060087

**Published:** 2018-06-05

**Authors:** Eric B. Sobotik, Jason T. Lee, Scott Hagerman, Gregory S. Archer

**Affiliations:** 1 Department of Poultry Science, Texas A&M University—College Station, TX 77843, USA; esobotik12@gmail.com (E.B.S.); jtlee@tamu.edu (J.T.L.); 2 Chemi Nutra, LLC—Austin, TX 78758, USA; shagerman@cheminutra.com

**Keywords:** broiler, phosphatidic acid, growth, yield

## Abstract

**Simple Summary:**

Improving feed conversion while increasing growth is a goal of any broiler nutrition program. The use of feed additives to obtain this goal has increased in recent years. However, increased growth in broilers has resulted in meat quality issues such as woody breast and white striping. In humans, utilizing phosphatidic acid (PA) in the diet has demonstrated increased lean muscle formation. If PA could be used in poultry, it may allow for increased growth without the pitfalls of poor meat quality.

**Abstract:**

The use of feed additives to improve feed conversion while increasing growth is the goal of any broiler nutrition program. Therefore, it is important to evaluate potential feed additives not only for increased performance, but also for any negative attributes. A study was conducted to evaluate the effects of feeding phosphatidic acid (PA) to broiler chickens. Two experiments were conducted using exercise in conjunction with PA (Experiment 1(E1)) and administering PA at different inclusion rates in the diet (Experiment 2 (E2)); LowPA (5 mg/bird/day), MidPA (10 mg/bird/day), HighPA (15 mg/bird/day), and control (CON). All birds were weighed bi-weekly during the experiments to obtain average pen weights and feed conversion ratios (FCRs). At the end of the experiments, eight birds per pen were processed to evaluate carcass traits and breast yield. In E1, exercise did not affect growth, feed conversion or processing traits (*p* > 0.05). However, PA supplementation did increase growth, carcass and breast weight, and carcass and breast yields (*p* < 0.05). In E2, differences (*p* < 0.05) in live bird weights between the control birds (1.65 kg) and all PA treatments (pooled mean: 1.73 kg) began at 28 days; however, only the LowPA carried that effect (*p* = 0.05) through to the conclusion of the trial (3.55 vs. 3.81 kg). Overall, LowPA (1.64) and MidPA (1.69) had lower (*p* < 0.05) FCRs than the CON treatment (1.74). Increased growth observed in live bird weights in the LowPA translated to increased (*p* < 0.05) overall carcass weights (2.78 vs. 2.99 kg) and specifically breast filet weights (0.69 vs. 0.76 kg). Yields did not differ (*p* > 0.05), but with the increased weight feeding LowPA resulted in more total breast meat. Phosphatidic acid did not affect (*p* > 0.05) woody breast or white striping. In conclusion, dietary PA improved FCR, increased live bird weights, and increased breast fillet weight without increased incidence of white striping. These results indicate that feeding PA may increase production efficiency in broilers.

## 1. Introduction

The consumption of poultry meat products has continued to increase due to their low price, high nutritional value, absence of a religious effect, and suitability for processing [1]. Throughout the poultry industry, there have been tremendous improvements in growth rates and breast meat yield, resulting in substantial increases in commercial meat production. Nonetheless, these advances are associated with several significant implications in the quality of the meat obtained [2]. The white striping (WS) condition present in broiler breast fillets is characterized by the appearance of white striations running parallel to the muscle fibers on the surface of the pectoralis major muscle [3]. For consumers, visual appearance is the primary and most important attribute to assess the quality of a meat product in a sealed package. Thus, the presence of any condition negatively affecting the visual appearance of a product can influence the purchase decision, leading to potential economic loss [3]. In addition to product appearance, the presence of white striping can become a hindrance, in regard to further processing, leading to a reduction in the water holding capacity of a meat product during processing or storage [4], or poor product cohesion related to immaturity of intramuscular connective tissue [5].

Skeletal muscle mass and muscle fiber vary in size according to physiological and pathological conditions. An increase in muscle mass, muscle growth or hypertrophy occurs during development and in response to mechanical overload or exercise [6]. Mechanical stimuli play a significant role in the regulation of skeletal muscle mass [7]. The mammalian target of rapamycin (mTOR), a protein kinase, determines the mechanical regulation of muscle mass [7]. It has been shown that the activation of mTOR signaling is sufficient to induce an increase in muscle fiber size [7]. The mechanical activation of mTOR signaling involves phospholipase D (PLD) and the lipid second messenger phosphatidic acid (PA). Phosphatidic acid (PA) is a diacyl-glycerophospholipid, in which two fatty acids and a phosphate group are covalently bonded to a glycerol molecule through ester linkages. PA can act as a signaling lipid, is a precursor for the biosynthesis of other lipids, and is a major constituent of cell membranes. Intracellular concentrations of PA can increase as a result of mechanical stimuli. During mechanical stimulation, PA can activate mTOR signaling via a P13K- and ERK-independent mechanism, bind to the FKBP12-rapamycin binding (FRB) domain of mTOR, and directly activate mTOR kinase activity in vitro [7]. Hence, a mechanically induced increase in PA could lead to enhanced binding of PA and mTOR, resulting in the activation of mTOR signaling and ultimately muscle growth [7]. It has been demonstrated that exogenous PA can stimulate the mTOR pathway via its activation of the substrate S6 kinase [8,9]. However, the binding of PA to S6 kinase may occur independently of mTOR [10], suggesting that PA may augment the signaling response when mTOR is activated by exercise.

While PA can be synthesized from a variety of reactions via multiple reactants, it is not clear if other precursors (i.e., glycerol-3-phosphate (G3P), LPA, or diacylglycerol (DAG)), or the addition of head groups to the PA molecule (i.e., phosphatidylcholine (PC), phosphatidylserine (PS), phosphatidylethanolamine (PE), or phosphatidylinositol (PI)), have a similar ability to activate mTOR signaling. Furthermore, different sources of PA (e.g., soy, egg) can have varying degrees of unsaturated or saturated fatty acid chains which can influence the behavior of PA. Foster [11] suggested that two saturated fatty acids will promote storage, but one saturated and one unsaturated fatty acid will promote signaling. In addition to controlling the rate of protein synthesis, mTOR also regulates transcriptional changes in response to a variety of conditions, such as cell cycle progression, actin organization, autophagy, synaptic plasticity, memory, and learning [12]. In turn, both intrinsic and extrinsic factors may have a direct impact on the mTOR pathway, due to the number of processes modulated by mTOR [12]. The mTOR signaling network is wired to growth factor signaling via the insulin/insulin-like signaling system (IIS) [13]. Decreased IIS/TOR signaling activity has been associated with an increased resistance to certain types of stress, indicating the pathway plays a vital role in the adaption to different stress conditions [14,15]. The IIS/mTOR signaling is positively correlated with muscle growth. Therefore, IIS and mTOR pathways play an important role in hypertrophic muscle accumulation [16].

The objective of this study was to evaluate the effect of dietary PA supplementation on broiler chicken growth and meat yield. To accomplish this, two experiments were conducted. The first examined if exercise was needed along with PA supplementation to increase muscle growth in broiler chickens. The second experiment evaluated different inclusion rates of PA in the feed. It was hypothesized that PA supplementation will increase muscle growth and yield, and that exercise will increase each of these factors.

## 2. Materials and Methods

### 2.1. Experiment 1 Exercise Trial: Animals and Husbandry

A total of 300 male Cobb 500 broilers were used in this experiment. Birds were equally housed at 25 birds per pen across a total of 12 pens (0.91 m × 2.74 m). Birds were randomly assigned to each pen; however, initial pen weights were equalized (1.04 kg). Each pen was lined with used pine shavings equipped with one bell feeder and a nipple drinking system. A 2 × 2 factorial design was used in this experiment in which pens were assigned at random to one of two dietary treatments: control diet (CON) or control diet with Mediator^®^ Phosphatidic Acid (Mediator^®^ PA, Chemi Nutra, Austin, TX, USA) added at a rate so birds consumed 15 mg/bird/day (PA) and one of two exercise treatments (Exercise or No Exercise). Half of the birds were subjected to “exercise” three times a day daily from the start of the experiment until the end of the experiment. The exercise consisted of lifting the birds to cause wing flapping for a duration of 30 s three times daily. Birds were fed a three-phase diet consisting of a starter (Days 0–14, crumble), grower (Days 14–28, pellet), and finisher 1 (Days 28–42, pellet) presented in Table 1. All feed was pelleted at 185 °F. Birds were allowed ad libitum access to feed and water. A photoperiod of 24L:0D was maintained to 10 days of age and then reduced to 20L:4D for the remainder of the study. Birds were raised to 42 days of age. The birds were managed according to the guidelines set forth in the Guide for the Care and Use of Agricultural Animals in Research and Teaching (FASS, 2010), and all procedures were approved by the USDA-ARS animal care committee (IACUC 2017-0179).

### 2.2. Experiment 2 Dose-Response Trial: Animals and Husbandry

A total of 648 male Cobb 500 broilers were used in this experiment. Birds were equally housed at 18 birds per pen across a total of 36 pens (0.91 m × 2.74 m). Birds were randomly assigned to each pen; however, initial pen weights were equalized (0.79 kg). Each pen was lined with used pine shavings equipped with one bell feeder and a nipple drinking system. Pens were blocked within and the treatments were assigned at random to one of four dietary treatments: control diet (CON) or control diet with Mediator^®^ Phosphatidic Acid (Mediator^®^ PA, Chemi Nutra, Austin, TX, USA) added at a rate so birds consumed 5 mg/bird/day (PALow), 10 mg/bird/day (PAMid) or 15 mg/bird/day (PAHigh). Birds were fed a three-phase diet consisting of a starter (Days 0–14, crumble), grower (Days 14–28, pellet), finisher 1 (Days 28–42, pellet), and finisher 2 (42–49, pellet) presented in Table 1. All feed was pelleted at 185 °F. Birds were allowed ad libitum access to feed and water. A photoperiod of 24L:0D was maintained to 10 days of age and then reduced to 20L:4D for the remainder of the study. Birds were raised to 49 days of age. The birds were managed according to the guidelines outlined in the Guide for the Care and Use of Agricultural Animals in Research and Teaching (FASS, 2010), and all procedures were approved by the USDA-ARS animal care committee (IACUC 2017-0179).

### 2.3. Growth, Feed Conversion, Processing Parameters

The birds in each pen were weighed at day 0, 14, 28, and day 42 in Experiment 1 and also on day 49 in Experiment 2. Body weight gain was calculated by subtracting day 0 weight from each weigh day. Feed was weighed before it was added to the feeder in each pen and residual feed was weighed back on weigh days so that feed intake could be calculated. Feed conversion ratio (FCR) was calculated by dividing the total feed intake per pen by the total body weight gain per pen and was corrected for mortality (mortality was weighed and added to final pen weights). At the end of each experiment following 8 hours of feed withdrawal, 8 birds per pen were processed and deboned for carcass and breast yields. In Experiment 2, following breast weight determination, fillets were palpated and visually inspected for the presence of woody breast and white striping. Woody breast was scored using Table 2 and white striping using the method found in Kuttappan et al. [3].

### 2.4. Statistical Analysis

All data was analyzed via One-Way ANOVA using the GLM model (SPSS Software, IBM, Armonk, NY, USA) with treatment means deemed significantly different at *p* ≤ 0.05. Treatment means that were determined to be significant were further separated using Duncan’s Multiple Range Test. All of the assumptions were tested (Shapiro–Wilk test for normality, Levene’s test for homogeneity of variance). No transformations were needed to meet assumptions.

## 3. Results

### 3.1. Experiment 1: Exercise Trial

The live bird weights for Experiment 1 are presented in Table 3. No interaction between feed type and exercise were observed at any time during the growout (*p* > 0.05). There was also no effect of exercise on bird weights at any time during Experiment 1 (*p* > 0.05). However, the addition of PA in the diets did affect bird weights during Experiment 1 (*p* < 0.05). All birds started at the same average weight, but by day 14 the control birds weighed less than the PA fed birds (*p* < 0.05); this continued for the remainder of the study. 

The data for feed conversion in Experiment 1 is presented in Table 4. There was no interaction effect or differences in either main effect observed with respect to feed conversion (*p* > 0.05). The processing data for Experiment 1 are presented in Table 5. There was no interaction or exercise effect on any of the processing data in Experiment 1 (*p* > 0.05). However, inclusion of PA in the diet did affect processing data (*p* < 0.05). The birds fed PA had greater carcass and breast weights and larger carcass and breast yields than the birds fed the control diet.

### 3.2. Experiment 2: Dose-Response Trial

The live bird weights for Experiment 2 are presented in Table 6. The inclusion of PA in the diet at any level did not affect live bird weight until day 28. At day 28, all PA treatments weighed more than the control birds (*p* < 0.05). However, on day 41 and 49 only the PALow birds weighed more than the control birds (*p* < 0.05). The PAMid and PAHigh birds were intermediate of the other two treatments. The data for feed conversion in Experiment 2 is presented in Table 7. The PALow treatment had better feed conversion than all other treatments through the first 14 days of the trial (*p* < 0.05). Through the first 28 days of the trial the PALow treatment continued to have better feed conversion than the PAHigh and control birds (*p* < 0.05). The PAMid treatment was intermediate of the PALow and other two treatments through the first 28 days. There was no difference in feed conversion between any treatments after 41 days (*p* > 0.05). However, overall the PALow treatment had better feed conversion than the PAHigh and control treatments (*p* < 0.05) with the PAMid treatment being intermediate. The processing data for Experiment 2 are presented in Table 8. The PALow treatment had greater (*p* < 0.05) carcass weights than the control treatment with the PAMid and PAHigh treatments being intermediate. The PALow and PAMid treatments had greater (*p* < 0.05) breast weights than the control treatment with the PAHigh treatment being intermediate. There were no differences between any treatments in woody breast or white stripping scores or in carcass or breast yield (*p* > 0.05).

## 4. Discussion

The hypothesis that feeding PA along with exercise would result in increased growth specifically in the breast muscle was rejected. The addition of exercise did not increase the growth or meat yield in Experiment 1. However, increases were observed in birds receiving dietary PA. Experiment 2 also observed an increase in growth and meat yield at the low inclusion rate. This does not agree with the research in humans [17,18,19], which observed that exercise in conjunction with dietary PA increased muscle mass. In the human studies it is thought that PA and exercise work together to increase muscle growth, as PA is a signaling lipid in the mTOR pathway, and is increased in mechanical stimulation during exercise [7]. However, it has also been demonstrated that exogenous supplied PA can result in stimulation of the mTOR pathway without exercise [8,9] so it is possible that exercise is not needed in poultry to achieve increased muscle growth.

In addition, the method used to exercise birds during this study likely resulted in stress. Negative attributes associated with stress, such as decreased growth and feed conversion have been well documented [20], thus, counteracting any positive effects resulting from the combination of exercise and PA. This is supported by the results observed in Experiment 2 which indicate increases in growth and meat yields with solely dietary inclusion of PA without exercise

No differences were observed in the woody breast or white striping scores in Experiment 2. This is important, as the severity of white striping and woody breast have increased in recent years. White striations characterize white striping parallel to muscle fibers in which muscle fiber degeneration takes place with infiltration of fat and connective tissue [21,22,23]. Woody breast is characterized by pale broad areas of substantial hardness accompanied with white striations [24]. Woody breast also results in hard, rigid fillets in which muscle fiber degeneration takes place with infiltration of connective tissue [24,25]. Sihvo [25] concluded that fast growth rate, along with increased breast meat yield, plays a significant role in the development of woody breast in chicken. Higher growth rates are also associated with greater incidence of white striping in poultry [21]. With evidence indicating that both woody breast and white striping are a result of fast growth and muscle degeneration, it is possible that utilizing PA to stimulate the mTOR pathway could be the reason this study observed increased growth and meat yield. It is possible that stimulating muscle growth via the mTOR pathway [7] somehow counteracts or prevents the muscle degeneration observed in white striping and woody breast, although this requires further research to confirm.

In Experiment 2, the high inclusion rate did not see the same increased growth that it did in Experiment 1. It is possible that other environmental factors may affect high levels of dietary PA to increase growth in poultry. An example is stress. Stress affects the mTOR signaling pathway [13]. It is possible that there were external stressor differences, such as temperature that altered the effect of PA at different inclusion rates. This, however, merits further research to determine if this hypothesis is true. The mTOR pathway is also involved in immune function [26] and gut health [27] which are two factors not measured in this study but merit investigation.

## 5. Conclusions

This was the first study to our knowledge using PA in the diet of broiler chickens. The results of this study demonstrate that including PA in the diet of broilers can increase growth and meat yield without increasing meat quality issues such as white striping and woody breast. Utilizing PA in the feed can increase production efficiency in broilers chickens; however, other factors such as how PA supplementation affects things like gut health and immune function, as well as the effects of environmental stressors, need to be further investigated.

## Figures and Tables

**Table 1:** Diet formulations fed to broilers in Experiments 1 and 2.

		Starter (%)	Grower (%)	Finisher 1 (%)	Finisher 2 (%)
Corn		58.15	64.24	69.11	71.18
Soybean		35	29.29	24.45	22.16
Dl-met98		0.29	0.25	0.2	0.22
Lysine hcl		0.17	0.16	0.13	0.2
L-threonine 98.5%		0.06	0.05	0.04	0.07
Soy oil		2.65	2.56	2.86	2.9
Limestone		1.44	1.32	1.22	1.22
Biofos 16/21p		1.56	1.4	1.24	1.26
Salt		0.44	0.35	0.24	0.16
Sodium bicarb		0	0.12	0.27	0.38
Tamu trace minerals ^1^		0.05	0.05	0.05	0.05
Tamu vitamins ^2^		0.15	0.15	0.15	0.15
Salinomycin		0.05	0.05	0.05	0.05
Calculated nutrient concentration					
Protein	PCT *	22.29	20.00	18.00	17.16
Crude fat	PCT	5.19	5.27	5.70	5.80
Calcium	PCT	0.90	0.82	0.74	0.74
Phosphorus	PCT	0.71	0.66	0.61	0.60
Av phosphate	PCT	0.45	0.41	0.37	0.37
ME POULTRY kcal/kg	KCAL/KG	3047.12	3101.88	3168.23	3190.18
Methionine	PCT	0.62	0.55	0.48	0.49
Tsaa **	PCT	0.99	0.89	0.79	0.78
Lysine	PCT	1.32	1.16	1.00	0.99
Threonine	PCT	0.89	0.79	0.70	0.69
Sodium	PCT	0.19	0.19	0.19	0.19

^1^ Vitamin premix added at this rate yields 22,045 IU vitamin A, 7716 IU vitamin D3, 91 IU vitamin E, 0.04 mg B12, 11.9 mg riboflavin, 91.8 mg niacin, 40.4 mg d-pantothenic acid, 261.1 mg choline, 2.9 mg menadione, 3.50 mg folic acid, 14.3 mg pyroxidine, 5.87 mg thiamine, and 1.10 mg biotin/kg diet. ^2^ Trace mineral premix added at this rate yields 60.0 mg manganese, 60 mg zinc, 60 mg iron, 7 mg copper, 0.4 mg iodine, a minimum of 6.27 mg calcium, and a maximum of 8.69 mg calcium/kg diet. The carrier was calcium carbonate and the premix contained less than 1% mineral oil. * Percent (%). ** Total sulfur amino acid.

**Table 2:** Guidelines for woody breast scoring.^1^

Normal (0)—Flexible throughout
Mild (1)—Hardness mainly in cranial region, flexible at caudal region
Moderate (2)—Hardness throughout with some flexibility in mid to caudal region
Severe (3)—Very hard, rigid throughout fillet

^1^ Anything in between that was indistinguishable to be one of the defined numbers an added or subtracted score of 0.50 was included to further classify the degree of woody breast.

**Table 3:** Average live bird weights (kg) during growout in Experiment 1.

Treatment	Day 0	Day 14	Day 28	Day 42
Control/No Exercise	0.042	0.406	1.554	2.875
Control/Exercise	0.042	0.407	1.547	2.897
PA/No Exercise	0.042	0.419	1.586	2.972
PA/Exercise	0.042	0.436	1.640	3.025
Pooled SEM *	0.001	0.005	0.014	0.030
Main Effect Feed				
Control	0.042	0.407 ^a^	1.551 ^a^	2.885 ^a^
PA	0.042	0.427 ^b^	1.613 ^b^	2.999 ^b^
Main Effect Exercise				
No Exercise	0.042	0.412	1.570	2.924
Exercise	0.042	0.421	1.594	2.962
*p* value Feed Main Effect	1.00	0.011	0.008	0.048
*p* value Exercise Main Effect	1.00	0.170	0.189	0.500
*p* value Interaction	1.00	0.184	0.104	0.748

^ab^ Differing superscripts within column indicate significant differences *p* < 0.05. * Standard Error of the Means.

**Table 4:** Feed conversion ratio during growout in Experiment 1.

Treatment	FCR Day 1–14	FCR Day 1–28	FCR Day 1–42
Control/No Exercise	1.288	1.432	1.667
Control/Exercise	1.183	1.432	1.735
PA/No Exercise	1.221	1.443	1.701
PA/Exercise	1.222	1.433	1.728
Pooled SEM *	0.061	0.022	0.039
Main Effect Feed			
Control	1.235	1.432	1.701
PA	1.222	1.438	1.715
Main Effect Exercise			
No Exercise	1.254	1.438	1.684
Exercise	1.203	1.432	1.731
*p* value Feed Main Effect	0.790	0.743	0.752
*p* value Exercise Main Effect	0.329	0.749	0.292
*p* value Interaction	0.319	0.779	0.635

No statistical differences were observed. FCR = feed conversion ratio. * Standard Error of the Means.

**Table 5:** Processing data: carcass weight (kg), breast weight (g), carcass Yield (%), and breast yield (%) in Experiment 1.

Treatment	Carcass Weight	Breast Weight	Carcass Yield	Breast Yield
Control/No Exercise	2.331	574.9	76.8	24.7
Control/Exercise	2.221	537.7	76.5	24.1
PA/No Exercise	2.310	581.9	76.6	25.2
PA/Exercise	2.321	567.1	77.8	24.4
Pooled SEM *	0.015	5.5	0.2	0.1
Main Effect Feed				
Control	2.270 ^a^	554.1 ^a^	76.5 ^a^	24.4 ^a^
PA	2.319 ^b^	578.1 ^b^	77.2 ^b^	24.9 ^b^
Main Effect Exercise				
No Exercise	2.314	574.3	76.7	24.8
Exercise	2.275	557.8	77.1	24.5
*p* value Feed Main Effect	0.046	0.013	0.058	0.033
*p* value Exercise Main Effect	0.104	0.081	0.194	0.196
*p* value Interaction	0.073	0.413	0.996	0.767

^ab^ Differing superscripts within column indicate significant differences *p* < 0.05. * Standard Error of the Means.

**Table 6:** Average live bird weights (kg) during growout in Experiment 2.

Treatment	Day 0	Day 14	Day 28	Day 41	Day 49
PALow	0.044	0.420	1.744 ^a^	3.159 ^a^	3.818 ^a^
PAMid	0.044	0.415	1.723 ^a^	3.107 ^ab^	3.702 ^ab^
PAHigh	0.044	0.414	1.725 ^a^	3.057 ^ab^	3.685 ^ab^
Control	0.044	0.407	1.659 ^b^	2.985 ^b^	3.553 ^b^
Pooled SEM *	0.000	0.004	0.011	0.023	0.037
*p* value Treatment Main Effect	0.60	0.59	0.02	0.05	0.05

^ab^ Differing superscripts within column indicate significant differences *p* < 0.05. * Standard Error of the Means.

**Table 7:** Feed conversion ratio during growout in Experiment 2.

Treatment	FCR Day 1–14	FCR Day 1–28	FCR Day 1–41	FCR Day 1–49
PALow	1.152 ^a^	1.374 ^a^	1.559	1.649 ^a^
PAMid	1.289 ^b^	1.402 ^ab^	1.579	1.694 ^ab^
PAHigh	1.309 ^b^	1.434 ^b^	1.605	1.732 ^b^
Control	1.352 ^b^	1.452 ^b^	1.609	1.741 ^b^
Pooled SEM *	0.018	0.011	0.009	0.012
*p* value Treatment Main Effect	<0.001	0.04	0.09	0.01

^ab^ Differing superscripts within column indicate significant differences *p* < 0.05. * Standard Error of the Means.

**Table 8:** Processing data: carcass weight (kg), breast weight (kg), woody breast score, white striping score, carcass yield (%), and breast yield (%) in Experiment 2.

Treatment	Carcass Weight	Breast Weight	Woody Breast Score	White Striping Score	Carcass Yield	Breast Yield
PALow	2.991 ^a^	0.769 ^a^	1.36	1.13	79.4	25.6
PAMid	2.887 ^ab^	0.729 ^a^	1.25	1.08	79.5	25.2
PAHigh	2.870 ^ab^	0.727 ^ab^	1.20	1.02	79.2	25.3
Control	2.783 ^b^	0.693 ^b^	1.11	1.00	79.4	24.9
Pooled SEM *	0.029	0.011	0.05	0.03	0.1	0.2
*p* value Treatment Main Effect	0.05	0.05	0.35	0.48	0.62	0.37

^ab^ Differing superscripts within column indicate significant differences *p* < 0.05. * Standard Error of the Means.
